# *Phyllanthus Niruri* Standardized Extract Alleviates the Progression of Non-Alcoholic Fatty Liver Disease and Decreases Atherosclerotic Risk in Sprague–Dawley Rats

**DOI:** 10.3390/nu9070766

**Published:** 2017-07-18

**Authors:** Raghdaa Hamdan Al Zarzour, Mariam Ahmad, Mohd. Zaini Asmawi, Gurjeet Kaur, Mohammed Ali Ahmed Saeed, Majed Ahmed Al-Mansoub, Sultan Ayesh Mohammed Saghir, Nasiba Salisu Usman, Dhamraa W. Al-Dulaimi, Mun Fei Yam

**Affiliations:** 1 Discipline of Pharmacology, School of Pharmaceutical Sciences, Universiti Sains Malaysia, Penang 11800, Malaysia; amzaini@usm.my (M.Z.A.); madjed_25@yahoo.fr (M.A.A.-M.); sultan_a1976@yahoo.com (S.A.M.S.); nasibausman@gmail.com (N.S.U.); dhamora@yahoo.com (D.W.A.-D.); yammunfei@yahoo.com (M.F.Y.); 2 Institute for Research in Molecular Medicine (INFORMM), Universiti Sains Malaysia, Penang 11800, Malaysia; gurjeet@usm.my; 3 Discipline of Pharmaceutical Chemistry, School of Pharmaceutical Sciences, Universiti Sains Malaysia, Penang 11800, Malaysia; mohali141@yahoo.com

**Keywords:** *Phyllanthus niruri*, non-alcoholic fatty liver disease (NAFLD), insulin resistance, atherosclerosis, α-glucosidase, pancreatic lipase, cholesterol micellization, ellagic acid, phyllanthin

## Abstract

Non-alcoholic fatty liver disease (NAFLD) is one of the major global health issues, strongly correlated with insulin resistance, obesity and oxidative stress. The current study aimed to evaluate anti-NAFLD effects of three different extracts of *Phyllanthus niruri* (*P. niruri*)*.* NAFLD was induced in male Sprague–Dawley rats using a special high-fat diet (HFD). A 50% methanolic extract (50% ME) exhibited the highest inhibitory effect against NAFLD progression. It significantly reduced hepatomegaly (16%) and visceral fat weight (22%), decreased NAFLD score, prevented fibrosis, and reduced serum total cholesterol (TC) (48%), low-density lipoprotein (LDL) (65%), free fatty acids (FFAs) (25%), alanine aminotransferase (ALT) (45%), alkaline phosphatase (ALP) (38%), insulin concentration (67%), homeostatic model assessment of insulin resistance (HOMA-IR) (73%), serum atherogenic ratios TC/high-density lipoprotein (HDL) (29%), LDL/HDL (66%) and (TC–HDL)/HDL (64%), hepatic content of cholesterol (43%), triglyceride (29%) and malondialdehyde (MDA) (40%) compared to a non-treated HFD group. In vitro, 50% ME of *P. niruri* inhibited α-glucosidase, pancreatic lipase enzymes and cholesterol micellization. It also had higher total phenolic and total flavonoid contents compared to other extracts. Ellagic acid and phyllanthin were identified as major compounds. These results suggest that *P. niruri* could be further developed as a novel natural hepatoprotective agent against NAFLD and atherosclerosis.

## 1. Introduction

Non-alcoholic fatty liver disease (NAFLD) is the hepatic manifestation of metabolic syndrome, which is highly associated with several metabolic disorders such as type 2 diabetes mellitus (T2DM), insulin resistance (IR) and hyperlipidemia [1,2]. In the absence of excessive alcohol ingestion, obesity is one of the major etiological factors of NAFLD, which is characterized by an accumulation of triglycerides inside the liver cells to a percentage exceeding 5% of liver weight [3], with the same histopathological manifestations of an alcoholic liver injury [4]. During the last two decades, its prevalence has reached worrying proportions as it has come to affect 25.2% of the people worldwide [5], including 20–30% of adults and 3–10% of children in Western countries [6]. NAFLD was reported as the main factor in many of the morbidities related to liver diseases [7]. It causes various types of liver injuries, including simple hepatic fat accumulation, which may occur alone or associated with steatohepatitis, known as non-alcoholic steatohepatitis (NASH), with or without fibrosis [8,9]. It was suggested by several studies that NAFLD is the key player in increasing the incidence of hepatocellular carcinoma [10]. The pathogenesis of NAFLD is multifactorial, but it is mainly attributed to insulin resistance and imbalance in lipids (uptake, hepatic synthesis and degradation) [9]. Recently, gut dysbiosis has also been linked with NAFLD [11].

Insulin resistance and oxidative stress are essential risk factors for NAFLD [12,13]. Oxidative stress promotes the production of reactive oxygen species (ROS) that stimulate an inflammatory process in hepatic tissues [14]. On the other hand, insulin resistance inhibits the antilipolytic activity of insulin in the adipose tissue and increases free fatty acids (FFAs) in the serum and liver, leading to mitochondrial dysfunction as well as cardiac fat accumulation [15].

Over the last decade, several noteworthy research initiatives have aimed at finding possible therapies to ameliorate the hepatic damage that accompanies NAFLD. The majority of pharmacological strategies include using antioxidant agents and insulin sensitizers, and also reducing the effect of dietary carbohydrate and fats by the inhibition of cholesterol micellization, pancreatic lipase and α-glucosidase [16,17,18].

Recently, a considerable number of studies has investigated natural phytochemicals as anti-NAFLD agents [19,20,21,22]. *P. niruri*, a herb found in South East Asia, has been traditionally used to treat many pathological conditions like dyspepsia, bronchitis, influenza, asthma, dysentery, tumours, diabetes, vaginitis and tuberculosis [23]. It was also used for its potent activity in the treatment of kidney stones and gallstones [24] and various liver disorders, particularly hepatitis and jaundice. Recent data showed that *P. niruri* had hepatoprotective properties against induced hepatitis in rats [25], and it has a therapeutic effect on T2DM, which is associated with hypercholesterolemia [26]. 

*P. niruri* is rich in flavonoids and phenolic compounds that are responsible for its potent antioxidant properties [27], which could play important roles in hepatoprotective activity [28,29]. However, to the best of our knowledge, no reports have been published regarding the therapeutic effects of *P. niruri* in the treatment of NAFLD. Thus, in this study we attempt to evaluate the potential role of *P. niruri* in suppressing and/or ameliorating the development of NAFLD induced by the oral administration of a high-fat diet (HFD) in rats as a model that reflects human NAFLD, using metformin as the positive control drug.

## 2. Materials and Methods

### 2.1. High-Fat Diet Preparation

A normal diet was purchased from Gold Coin Feed mills Sdn. Bhd., Penang, Malaysia (product code 702P), which contains crude protein (21–23%), crude fibre (5%), crude fat (3%), moisture (13%), ash (8%), calcium (0.8%) and phosphorus (0.6–1.0%). The high-fat diet was prepared by mixing the normal diet with 10% margarine (wt/wt), 10% ghee fat (wt/wt), 1% cholesterol and 0.5% cholic acid.

### 2.2. Extract Preparation

A specimen of *P. niruri* including whole plant was identified by Professor Mashhor Mansor from the Botany Department, School of Biological Sciences, Universiti Sains Malaysia (USM). A voucher herbarium specimen has been deposited at the Herbarium Unit (Voucher No. 11474), School of Biological Sciences, USM. A powder of dry whole plant of *P. niruri* was extracted by maceration using water (WE), 50% methanol in water (50% ME) and methanol (ME) respectively.

### 2.3. In Vivo Model

The NAFLD animal model was developed and studied using 36 male Sprague–Dawley (SD) rats (10 weeks of age), obtained from the Animal Research and Service Centre, USM. All experimental procedures were carried out after getting the approval from Animal Ethics Committee, Universiti Sains Malaysia (protocol No.: 2013/(90) (546)).

Animals were acclimatized for one-week and randomly assigned to six groups (*n* = 6). Group 1 was fed a normal diet for eight weeks and served as the normal control group (NC), while the five remaining groups (2–6) were fed a high-fat diet (HFD) for eight weeks. Oral gavage treatment started in week 4. Group 1was treated with distilled water (10 mL/kg body weight). Group 2 was the HFD group, which served as a negative control and received distilled water (10 mL/kg body weight); group 3 served as a positive control and received metformin (HFD + Met 500 mg/kg body weight) at 10 mL/kg body weight; groups 4–6 received 1000 mg/kg of *P. niruri* WE, 50% ME, and ME, respectively, at 10 mL/kg body weight

The same protocol was repeated for evaluating the dose-response relationship, using five groups. Group 1 was fed a normal diet and served as a normal control group (NC), while the four remaining groups were fed a high-fat diet (HFD) for eight weeks. Oral gavage treatment started in week 4. Group 1 was treated with distilled water (10 mL/kg body weight). Group 2 served as a negative control and received distilled water (10 mL/kg body weight); groups 3, 4, and 5 received 1000 mg/kg, 500 mg/kg, and 250 mg/kg, respectively, of the most active extract of *P. niruri* to evaluate the relative potency of the three different doses (1000 mg/kg, 500 mg/kg, and 250 mg/kg) at 10 mL/kg body weight.

### 2.4. Determination of Blood and Serum Biochemical Parameters

After eight weeks of HFD feeding, all the rats were fasted overnight and the body weight of each rat was determined. They were anesthetized with sodium pentobarbital (60 mg/kg intraperitoneal (i.p.)). Five millilitres of the blood were taken via cardiac puncture from each rat. Blood glucose level was determined using an automated blood glucose meter (Accu-chek Performa^®^, Roche Diagnostics, Mannheim, Germany). Peri-renal fats were isolated and weighed for the assessment of visceral adipose tissues. The serum was used to measure aspartate transaminase (AST), alanine transaminase (ALT), alkaline phosphatase (ALP), total bilirubin, creatinine, uric acid, urea, low-density lipoprotein (LDL), high-density lipoprotein (HDL), total cholesterol (TC), and triglycerides (TG) levels using the Olympus AU640™ multifunctional biochemistry analyser (laboratories of Gribbles, Penang, Malaysia). 

### 2.5. Determination of Insulin and Free Fatty Acids Concentrations

Insulin levels were determined using the commercial rat insulin ELISA kit (Elabscience Biotechnology Co., Ltd., Beijing, China). Serum free fatty acids (FFAs) values were measured using a fluorometric assay kit (Cayman Chemical, Ann Arbor, MI, USA) catalogue No. 700310.

### 2.6. Assessment of Atherosclerosis Risk Indexes

Recent data showed that NAFLD is one of the independent predictors of atherosclerosis [16]. Therefore, in order to identify high-risk cases of atherosclerosis and their effective therapeutic management, the predictor ratios that complement the lipid profile ratios such as atherogenic Castelli’s Risk Index I, Castelli’s Risk Index II, in addition to atherogenic coefficient (AC) were determined [30].

Atherogenic ratios were calculated based on the following formulas:

Castelli’s Risk Index-1 (CRI-I) = TC/HDL;(1)

Castelli’s Risk Index-2 (CRI-II) = LDL/HDL;(2)

Atherogenic Coefficient (AC) = (TC − HDL)/HDL.(3)

### 2.7. Assessment of Lipid Peroxidation, TG and TC Contents in Liver Tissues

Total protein content of liver tissues was determined using a Bradford assay kit (Sigma-Aldrich, San Jose, CA, USA). The MDA content was measured in the hepatic tissue homogenate by a thiobarbituric acid reaction [31]. Hepatic lipid was extracted according to Folch et al. [32]. The content of TG was determined using triglycerides colorimetric assay kit (Cayman Chemical, Ann Arbor, MI, USA), while TC content was determined using a commercial kit according to the manufacturer’s protocol (Thermo Fisher Scientific, Waltham, MA, USA).

### 2.8. Histopathological Examinations

For histological analysis, the liver specimen was taken from the largest hepatic lobe and fixed with formaldehyde 10% (*v*/*v*). Histopathological examinations were performed by an experienced pathologist using a blinded approach. The liver sections were stained with hematoxylin and eosin (H&E). The histological features were examined and grouped into four broad categories: steatosis, lobular inflammation, hepatocellular ballooning, and fibrosis. A score system was utilized to evaluate each feature. An overall score, namely the NAFLD activity score (NAS), was generated based on the individual scores of all the histological features [33]. 

### 2.9. Antioxidant Activity Tests

The antioxidant assays of the three extracts of *P. niruri* were carried out in triplicate at a concentration of 10 mg/mL. Absorbance was measured using a microplate reader (TECAN Infinite Pro^®^ M200, Mannedorf, Switzerland). Total phenolic content (TPC) of each extract was estimated using Folin-Ciocalteu reagent, according to the method of Kumaran and Joel Karunakaran [34]. Gallic acid was used as the standard. TPC was expressed as µg gallic acid equivalent/mg dry extract. Total flavonoid content (TFC) of each extract was measured using the aluminium chloride method [35]. Quercetin was used as a reference standard. TFC was expressed as µg quercetin equivalent/mg dry extract. DPPH scavenging activity was assessed according to the method developed by Al-Mansoub et al. [36]. Ascorbic acid was used as the reference standard. The scavenging activity was expressed as IC_50_. ABTS scavenging activity was performed using the method described by Al-Mansoub et al. [36]. Ascorbic acid was used as reference standard. Results were expressed as IC_50_. The ferric reducing antioxidant power of each extract (FRAP) was carried out by the method of Benzie and Strain [37]. All results were expressed as nmol Fe^+2^ equivalent/mg dry extract.

### 2.10. In Vitro Assay to Determine the Inhibitory Effects of the Most Active Extract on α-Glucosidase, Pancreatic Lipase and Cholesterol Micellization

The inhibitory activity of the most active extract against α-glucosidase was measured based on previous method described by Yusoff et al. [38] with minor modification. The results were expressed as values of IC_50_ which is defined as the concentration of the sample/acarbose required to inhibit 50% of α-glucosidase activity. Absorbance reading was taken using a microplate reader (TECAN Infinite Pro^®^ M200, Mannedorf, Switzerland). All analyses were done in triplicate. Six serial dilutions of each sample were used to obtain the IC_50_ values. The inhibitory effect of the active extract towards pancreatic lipase was assessed by fluorometric assay based on the method described by Zhang et al. [39,40], using orlistat as the standard. The results were expressed as IC_50_ which is defined as the concentration of either sample or orlistat required to inhibit 50% of pancreatic lipase activity. 

The inhibitory activity of most active extract of *P. niruri* against cholesterol micelle formation was carried out based on a previously reported method with minor modifications [41], using gallic acid as the positive control and a total cholesterol test kit for cholesterol determination.

### 2.11. Phytochemical Analysis by High-Performance Liquid Chromatography (HPLC)

To standardize the most active extract and further elucidate its chemical composition and active compounds, an HPLC analysis was performed. Stock solutions (1000 μg/mL) of two selected marker compounds (ellagic acid and phyllanthin) were prepared in methanol. The chromatographic separation was carried out using a Zorbax Eclipse Plus C18 column and HPLC device (Agilent, Santa Clara, CA, USA). Column specifications were 250 × 4.6 mm/5 μm particle size. The samples (10 μL) were eluted at a flow rate of 1 mL/min at a λ_max_ of 230 nm, with a gradient mobile phase comprising 0.2% aqueous acetic acid and acetonitrile. The lowest limit of detection (LOD) value for each marker compounds was determined by analysing the standard concentrations successively with a 2-fold dilution with methanol. The concentration with the smallest detectable peak at a noise-to-signal ratio of 3 was considered as the LOD value. Moreover, the lower linearity range limit at 83 noise-to-signal ratio of 10 was deemed as the limit of quantification (LOQ) [42]. Each working standard solution was injected (*n* = 5) to determine the precision of the method. The values were expressed as the coefficient of variation (CV%). Intra-day accuracy and precision analyses were carried out by injecting the working standard concentrations (*n* = 5) within one day, whereas the intra-day analyses were conducted by injecting the standard working concentrations once per day for five consecutive days (Murugaiyah and Chan, 2007). Three working standard concentrations of each selected marker compound were used to determine the percentage of recovery. The percentage of recovery was calculated for each analyte according to the equation below [43]:

Recovery (%) = A − B/C × 100(4)

where, A = amount of marker compound detected; B = original amount of the marker compound; C = amount of the marker compound spiked.

Ellagic acid and phyllanthin were quantified in *P. niruri* extract. The extract (1 mg/mL) was prepared in methanol for HPLC analysis. All the samples were filtered by 0.45 μm PTFE syringe filter. The selected marker compounds were quantified in triplicates using linear regression equations of the calibration curves.

### 2.12. Statistical Analysis

The results were expressed as means ± S.E.M. Statistical significant difference was examined using Statistical Package of Social Sciences (SPSS) program, version 20 (IBM Corp., Armonk, NY, USA). Differences between groups were evaluated using the one-way analysis of variance (ANOVA), followed by Dunnett’s post hoc test, and was considered significant when *p* < 0.05.

## 3. Results

### 3.1. P. niruri Reduced Hepatomegaly and Visceral Adiposity

**Figure 1:**
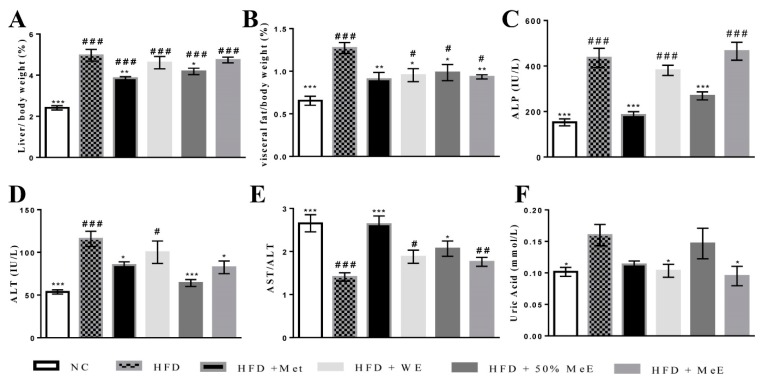
Effect of *P. niruri* extracts and metformin on liver weight and visceral fat ratios, serum liver enzymes and serum uric acid in high fat diet-fed rats. (**A**) Liver/body weight %; (**B**) visceral fat/body weight %; (**C**) ALP; Alkaline phosphatase; (**D**) ALT; Alanine aminotransferase; (**E**) AST/ALT; (**F**) serum uric acid. (Results expressed as mean ± SEM (*n* = 6). * *p* < 0.05, ** *p* < 0.01, *** *p* < 0.001 vs. HFD, and ^#^ *p* < 0.05, ^##^ *p* < 0.01, ^###^ *p* < 0.001 vs. NC group). NC; Normal control, HFD; High fat diet, Met; Metformin, WE; Water extract, MeE; Methanolic extract, SEM; Standard error mean.

The results revealed that the liver to body weight ratio in the HFD group was 51.4% higher than in the normal control (NC) group. Similarly, the visceral fat to body weight ratio was 48.6% higher in the HFD group compared to the NC group (*p* < 0.01), indicating that HFD rats developed hepatomegaly and visceral adiposity. These changes were reversed by metformin and 50% ME of *P. niruri*, both of which significantly inhibited the increase in liver weight compared with the HFD group by 22.5% (*p* < 0.01) and 15.8% (*p* < 0.05), respectively (Figure 1A), while the visceral fat weight was significantly reduced in groups treated with metformin, WE, 50% ME, and ME of *P. niruri* by 28.7%, 24.9%, 22.5%, and 26.5%, respectively, compared with the HFD group (Figure 1B). 

### 3.2. P. niruri Improved Abnormalities in Serum Indicators of NAFLD Rats

**Figure 2:**
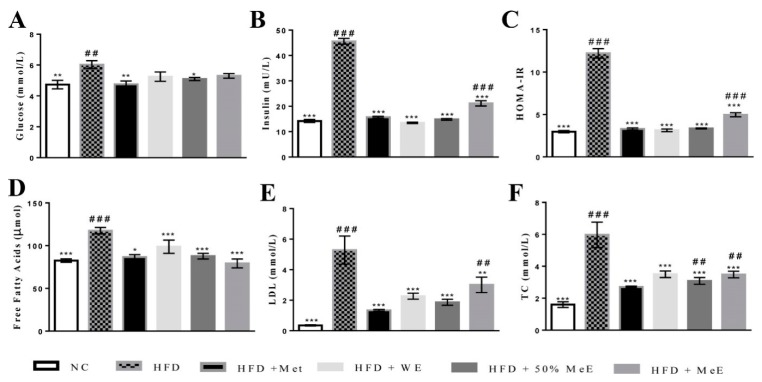
Effect of *P. niruri* extracts and metformin on serum levels of glucose, insulin, HOMA-IR, FFAs, LDL and TC in HFD-fed rats. (**A**) Serum levels of glucose; (**B**) serum levels of insulin; (**C**) serum levels of HOMA-IR; (**D**) serum levels of FFAs; (**E**) serum levels of LDL; (**F**) serum levels of TC. (Results expressed as mean ± SEM (*n* = 6). * *p* < 0.05, ** *p* < 0.01; *** *p* < 0.001 vs. HFD, and ^#^ *p* < 0.05; ^##^ *p* < 0.01; ^###^ *p* < 0.001 vs. NC group). HOMA-IR; homeostatic model assessment of insulin resistance, FFAs; Free fatty acids, LDL; Low density lipoprotein, TC; Total cholesterol.

To assess the development of NAFLD in the HFD model, the serum indicators correlated with NAFLD were measured. As shown in Figure 1 and Figure 2 and Table 1, levels of glucose, total cholesterol, LDL, CRI-I, CRI-II, AC, and uric acid were significantly increased by 21.5%, 73.1%, 93.3%, 65.9%, 92.7%, 90.2%, and 36.5%, respectively (all *p* < 0.01 except uric acid *p* < 0.05) in the HFD group when compared with the NC group. Moreover, liver enzymes including ALP and ALT levels were also rapidly doubled (both *p* < 0.01), with no significant effect on AST, while AST/ALT ratio was decreased by almost half in the HFD group as compared to the normal diet group. However, the changes induced by HFD in TG, HDL, AST, total bilirubin, creatinine and urea levels were not statistically significant. On the other hand, compared with the HFD group, treatment with metformin, WE, 50% ME, and ME of *P. niruri* has showed enhanced reduction in the levels of TC by 54.7%, 41.3%, 48.3%, and 41.6%, respectively. LDL was lowered by 74.65%, 57.13%, 64.68%, and 43%, respectively. CRI-II was reduced by 74.71%, 59.38%, 65.89%, and 41.43%, respectively. AC values were also decreased by 73.3%, 58.9%, 64.4%, and 36%, respectively. However, only the treatment with metformin and 50% ME of *P. niruri* exhibited significant additional effects including the reduction of glucose by 21.2% and 15.5%, respectively; CRI-I by 46.7% and 29.3%, respectively; ALP levels by 57.6% and 38.3%, respectively; as well as improving AST/ALT ratios by 48% and 43.3%, respectively. ALT was significantly decreased in rats treated with metformin by 26.5%, ME of *P. niruri* by 28.8% (both *p* < 0.05), and 50% ME 44.6% by (*p* < 0.01), whereas uric acid was significantly restored to normal value after treatment with WE and ME of *P. niruri* (*p* < 0.05).

### 3.3. P. niruri Decreased FFAs and Insulin Resistance

To evaluate the extent of insulin resistance in our model, we further measured the serum concentration of FFAs and insulin. Our results showed that eight weeks of oral administration of HFD has increased serum FFAs by 29.8% (*p* < 0.01), and serum insulin level by 68.8% (*p* < 0.01) and HOMA-IR by 75.7% (*p* < 0.01), indicating that rats the HFD developed distinct insulin resistance. However, treatment with metformin, WE, 50% ME, and ME of *P. niruri* markedly reduced FFAs by 26.2%, 15.9%, 25.3%, and 32.5%, respectively, restored insulin levels to normal, and ameliorated insulin resistance (Figure 2). 

### 3.4. P. niruri Reduced the Liver Content of TC, TG and Oxidative Stress Indicator

**Figure 3:**
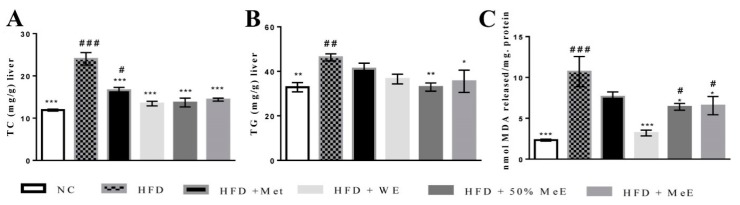
Effect of *P. niruri* extracts and metformin on Hepatic levels of TC, TG and MDA: (**A**) hepatic levels of TC; (**B**) hepatic levels of TG; (**C**) hepatic levels of MDA. (Results expressed as mean ± SEM (*n* = 6). * *p* < 0.05, ** *p* < 0.01, *** *p* < 0.001 vs. HFD, and # *p* < 0.05, ## *p* < 0.01, ### *p* < 0.001 vs. NC group). TG; triglycerides, MDA; malondialdehyde.

Fat accumulation and lipid peroxidation in liver plays an essential role in the development of NAFLD [15]. To study the effects of *P. niruri* extracts on NAFLD progress in HFD-fed rats, we measured the content of hepatic TC, TG and MDA. High-fat diet caused a significant lipid droplet accumulation. Our results showed considerable increase in hepatic content of triglycerides by 28.9%, total cholesterol by 50.5% and malondialdehyde (MDA) levels by 78.2% compared to the normal control group (all *p <* 0.01). However, hepatic cholesterol levels were significantly reduced by 30.9%, 44%, 43%, and 40.2% after four weeks of oral administration of metformin, WE, 50% ME, and ME of *P. niruri*, respectively (all *p* < 0.01). Moreover, notable decreases in the hepatic triglycerides levels were observed in animals treated with 50% ME by 28.9% (*p* < 0.01) and ME by 23.3% (*p* < 0.05). On the other hand, the results revealed that the increase in hepatic malondialdehyde has been significantly inhibited in groups treated with WE by 70% (*p* < 0.05), 50% ME by 40.1% (*p* < 0.01) and ME by 38.8% (*p* < 0.01) compared with the HFD group (Figure 3).

### 3.5. Effects of P. niruri on the Liver Histopathology

Liver biopsy is considered to be the gold standard in NAFLD diagnosis [44]. Therefore, to confirm the onset of NAFLD in the animal model, histological analysis was performed as described in other studies [45,46]. Steatosis, hepatocyte ballooning and inflammation were assessed and scores (Table 2) were given individually. The individual scores were added together to produce the overall NAFLD activity score (NAS).

**Figure 4:**
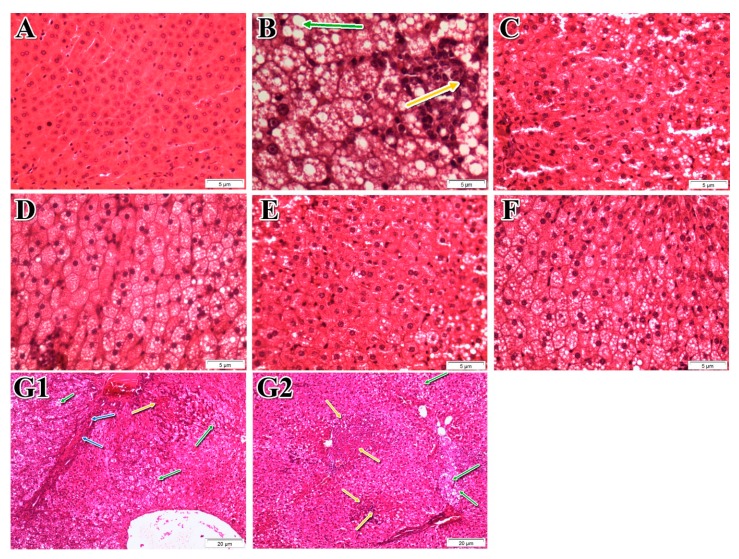
Effect of *P. niruri* extracts and metformin on the histological evaluation of liver biopsy. Typical liver tissue sections (400×) stained with haematoxylin and eosin (H&E). (**A**) NC group, showing normal tissue structure and architecture with no steatosis; (**B**) HFD group showed vesicular steatosis and inflammation (typical NAFLD); (**C**) HFD + metformin (500 mg/kg); (**D**) HFD + WE (1000 mg/kg); (**E**) HFD + 50% ME (1000 mg/kg) and (**F**) HFD + ME (1000 mg/kg). The severe hepatic damage (inflammation and fibrosis) in HFD rats is further shown in the Figure 4G1,G2 (100×) stained with haematoxylin and eosin (H&E), (**G1**) HFD group showed steatosis (green arrows) with fibrosis (blue arrows) (100×); (**G2**) HFD group showed lobular inflammation (yellow arrows) (100×).

Histological evaluation of liver samples from HFD group showed significant fat deposition with highest scores in steatosis, lobular multifocal portal inflammation, hepatocyte ballooning and fibrosis, significantly higher than those of the normal control group (*p* < 0.01). Hence, rats fed the high-fat diet developed definite NAFLD. Furthermore, the severe inflammation with fibrosis in the HFD-fed rats obviously confirmed the progression from NAFLD to NASH in the HFD group. However, the severity of the degenerative changes decreased following four weeks of metformin administration and all the *P. niruri* extracts, which resulted in significant suppression of steatosis, lobular inflammation, and NAS, but caused no considerable changes in hepatocyte ballooning (Figure 4 and Table 3).

### 3.6. In Vitro Tests for the 50% ME of P. niruri

Our biochemical and histological findings showed that 50% ME of *P. niruri* exhibited superior therapeutic effects among the treated groups. The 50% ME of *P. niruri* (2.5, 5, 10 and 20 mg/mL) exhibited inhibitory activity against the artificially prepared micelles of cholesterol in a dose-dependent manner by 4.27 ± 0.50%, 5.79 ± 2.10%, 12.03 ± 1.38%, and 12.91 ± 1.17%, respectively, while gallic acid (0.2 mg/mL), the standard compound, inhibited the cholesterol micelles’ formation by 23.05 ± 0.40%. The 50% ME of *P. niruri* also inhibited pancreatic lipase with IC_50_ value of 0.78 ± 0.05 mg/mL. However, it had less potent activity than orlistat which was 0.20 ± 0.01 µg/mL. An α-glucosidase inhibitory test demonstrated that 50% ME of *P. niruri* exhibited a strong inhibitory potential in a concentration-dependent manner. The percentage of inhibition ranged from 85.6% for the highest concentration (0.156 mg/mL) to 16.9% for the lowest concentration (0.0049 mg/mL). Moreover, the magnitude of inhibition of α-glucosidase enzyme activities by 50% ME of *P. niruri* was comparable to the standard inhibitor, acarbose. With reference to the IC_50_ values, acarbose IC_50_ was 4.29 ± 0.16 mg/mL, whereas the IC_50_ of 50% ME of *P. niruri* was 0.06 ± 00.0 mg/mL. The findings suggest that the inhibitory effects of 50% ME of *P. niruri* are more potent than acarbose.

### 3.7. The Effect of Different Doses of 50% ME of P. niruri on NAFLD

**Figure 5:**
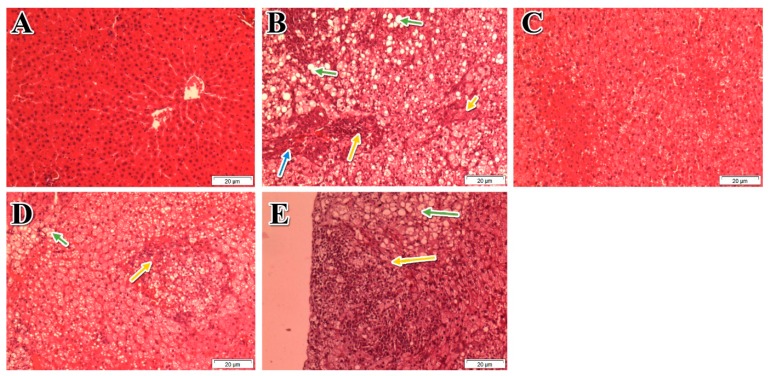
Microscopic examination of liver tissues stained with H&E after four-week treatment with different doses of 50% ME under 100× magnification. (**A**) Normal tissue structure; (**B**) steatosis, severe inflammation and fibrosis in HFD group; (**C**) marked reduction of NAFLD pathological manifestations at dose 1000 mg/kg of 50% ME; (**D**) moderate steatosis and inflammation at dose 500 mg/kg of ME50; (**E**) severe inflammation at the dose 250 mg/kg of ME 50 (green arrows: steatosis; yellow arrows: inflammation, blue arrows: fibrosis).

As shown in Table 4, all doses of 50% ME significantly decreased serum cholesterol, LDL, CRI-I, ALT, Insulin concentrations and HOMA-IR and increased the AST/ALT ratio. However, only the dose of 1000 mg/kg was able to significantly improve the histological parameters (Figure 5).

### 3.8. Total Phenolic Content (TPC), Total Flavonoid Content (TFC) and Anti-Oxidative Activities of P. niruri

The highest total phenolic and flavonoid contents were found in 50% ME of *P. niruri* with the highest antioxidant activities in DPPH and ABTS tests (Table 5).

### 3.9. Extract Standardization by HPLC Analysis

**Figure 6:**
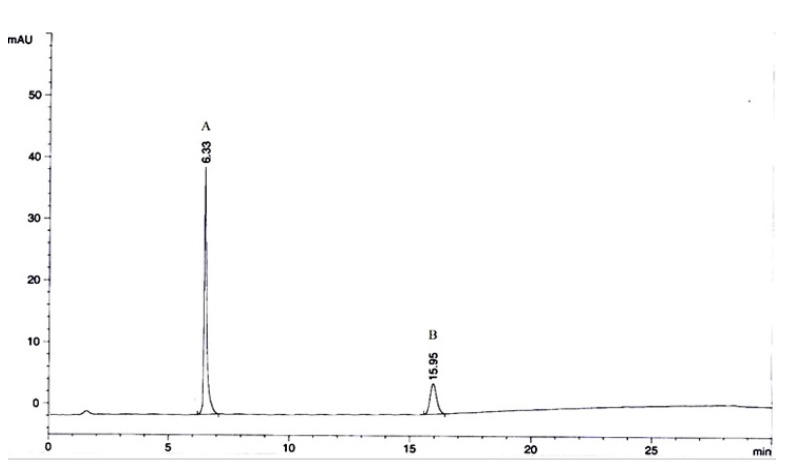
High performance liquid chromatography chromatogram of mixed standard compounds. A: ellagic acid; B: phyllanthin.

**Figure 7:**
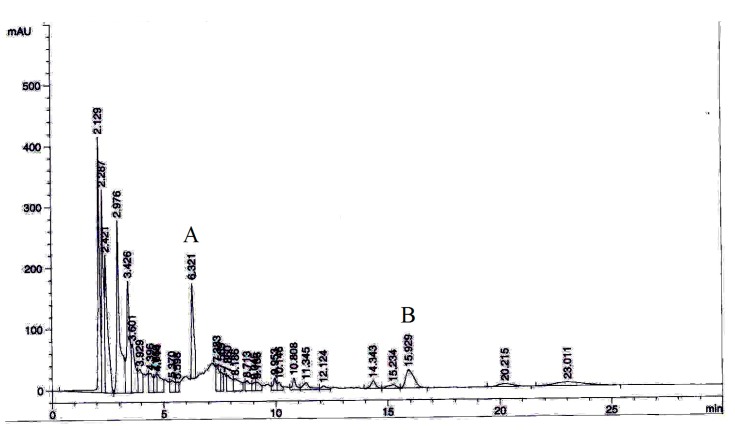
High performance liquid chromatography chromatogram of *P. niruri* extract. A: ellagic acid; B: phyllanthin.

All selected marker compounds showed good linear regression with high correlation coefficient values (*R^2^* ≥ 0.999) between the peak area and the concentration. LOD was 0.20 and 0.52 μg/mL, respectively; and LOQ values were 0.78 and 1.56 μg/mL, respectively, for ellagic acid and phyllanthin. Figure 6 shows a representative HPLC chromatogram of mixed standard compounds. Peaks appeared at 6.33 min for ellagic acid and at 15.95 min for phyllanthin. The recovery percentages for the selected marker compounds in *P. niruri* extract are presented in Table 6. Recovery values for the concentrations used ranged from 99.25% to 104.75% and from 100.04% to 102.65% for ellagic acid and phyllanthin, respectively. The recovery data were deemed satisfactory, leading to the conclusion that our extraction methodology did not cause substantial loss of those compounds. Data pertaining to the intra- and inter-day accuracy and precision obtained after analysing the selected marker compounds are summarised in Table 6. The accuracy values, expressed as percentages of the true values, were 95.90% and 104.94% for ellagic acid and phyllanthin, respectively. The corresponding precision values, expressed as CV%, totalled 0.18% and 4.78% for intra-day and inter-day analyses, respectively. This indicated that the method used was reliable and reproducible. Ellagic acid and phyllanthin were quantified in *P. niruri* using the HPLC technique described earlier. The HPLC chromatogram of *P. niruri* extract is shown in Figure 7. The HPLC-UV method used here was developed for simultaneous determination of ellagic acid and phyllanthin. Analysis revealed that that *P. niruri* contained several peaks at different retention times, demonstrating that multiple components were detected. However, the retention times of ellagic acid and phyllanthin were approximately 6.32–15.92 min. Both ellagic acid and phyllanthin were major compounds in *P. niruri* extract, with phyllanthin making up over 10% and ellagic acid comprising nearly 2% of the extract. We believe that the HPLC method used in this work can be used as a standard analytical method for the quantification of marker compounds in *P. niruri* extract.

## 4. Discussion

Sedentary lifestyle and overconsumption of high-calorie foods are the main reasons for central obesity and NAFLD [48]. The present study provides strong evidence of the anti-NAFLD activity of *P. niruri* in SD rats. Our optimized extract of *P. niruri* not only supressed hepatic fat accumulation but also blocked inflammation in NAFLD rats, thus preventing fibrosis and inhibiting simple steatosis from progressing to NASH. Additionally, rats treated with *P. niruri* exhibited significantly lower indicators of atherosclerosis than untreated rats. 

Our current in vivo model of NAFLD was successfully established and developed to NASH after feeding the rats with a HFD enriched with trans-fatty acids from margarine, and saturated fatty acids derived from animal fat [49,50]. The high increase in the weight of the liver and visceral fat in the HFD group is a prominent feature of NAFLD [51,52]. Furthermore, the hypercholesterolemia within HFD group was previously reported in 20–80% of human NAFLD cases [53], and a greater concentration of serum FFAs is correlated with disease severity [54]. Moreover, the significant increase in serum levels of ALP and ALT in addition to the elevated hepatic content of TG, TC and MDA in NAFLD rats is a strong indicator of liver damage and oxidative stress [55]. According to the “two hit” hypothesis of NAFLD, high levels of free fatty acids lead to lipogenesis and lipid accumulation in the liver during the first hit of NAFLD progress, whereas the presence of oxidative stress and lipid peroxidation represents the second hit [56]. More specifically, the accumulated fats in the liver induce the release of cytotoxic oxygen free radicals, which are associated with lipid peroxidation markers such as malondialdehyde (MDA) [57] that trigger inflammation and fibrosis [58,59]. In the current study, HFD group exhibited higher concentrations of glucose and hyperinsulinemia, elevated HOMA-IR values, and developed significant hepatic steatosis, inflammation and fibrosis. These results are in agreement with previous studies and further prove the ability of a HFD to cause rapid fat accumulation in the liver, with distinct elevation in insulin resistance (HOMA-IR values) [60]. The increase of insulin resistance in the HFD group would stimulate oxidative stress in the liver by releasing reactive oxygen species, causing hepatic lipid peroxidation and initiating inflammatory reactions that enhance fibrosis through the activation of hepatic stellate cell (HSC) [3,61].

In addition, the significant increase in serum uric acid (SUA) concentration in the HFD group led to the development of NAFLD and insulin resistance. It was reported that hyperuricemia is closely correlated with the severity of liver damage. Moreover, SUA can enhance inflammatory reactions and trigger oxidative stress in adipocytes, which in turn increases insulin resistance and induces more lipolysis in adipose tissue and greater oxidative stress in the liver cells [62]. Consequently, the reduction in SUA could improve NAFLD [63]. Similarly, the present findings suggest that decreasing the SUA level in WE- and ME-treated groups contributed to the suppression of NAFLD in these groups.

The considerable increase in serum atherogenic indexes indicates a higher risk of atherosclerosis in NAFLD rats. This is attributed to the sharp increase in serum levels of LDL, which is usually oxidized to form the peroxides that play a role in several atherosclerotic stages by their cytotoxic effects, leading to endothelial injury [64]. 

However, 50% ME of *P. niruri* was the most active among the three extracts of the plant. It prominently decreased visceral adipose tissue and liver weight, and blocked the progress of NAFLD by the reduction in hepatic steatosis and inflammation. In addition to preventing fibrosis, it improved liver function and decreased serum levels of glucose, insulin concentrations and HOMA-IR, indicating a significant decrease in insulin resistance. It was obvious from the results that *P. niruri* improved cross-talk between the liver and adipose tissues. This was reflected by the lower serum concentrations of TC, LDL and FFAs in NAFLD rats [65]. The 50% ME of *P. niruri* also supressed FFA-evoked de novo lipogenesis in the liver and decreased the hepatic content of cholesterol, TG, and MDA. As a result, the atherogenic ratios were evidently decreased. The results show that all doses (1000 mg/kg, 500 mg/kg, and 250 mg/kg) of 50% ME are able to significantly reduce atherogenic ratios, ALT, AST/ALT ratio, and suppress insulin resistance. On the other hand, liver weight, visceral adipose tissue weight and liver histology were only improved by the dose of 1000 mg/kg, indicating that this is the anti-NAFLD effective dose.

The anti-NAFLD effect of *P. niruri* is likely to result from the antioxidant activity shown by DPPH and ABTS tests. This is most likely attributable to phenolic compounds in all *P. niruri* extracts that inhibited oxidative stress by decreasing lipid peroxidation and improving insulin signalling and β-oxidation. These results are in agreement with many previous studies that explained the potent antioxidant activities of polyphenols in suppressing lipogenesis and inhibiting fatty acid synthesis in vitro [14]. The anti-NAFLD effect of 50% ME of *P. niruri* may also be due to ellagic acid, an active antioxidant that was a major compound in the extract. It was previously found to be responsible for anti-NAFLD effect of *Phyllanthus emblica* L. in vitro [66]. Ellagic acid was further reported to decrease de novo lipogenesis, inhibit TG esterification and enhance FFAs oxidation by increasing the expression of FFAs oxidative genes in the liver, leading to significant reduction of hepatic lipid accumulation [67]. It was suggested that this activity is due to the increase in energy expenditure by enhancing AMPK, the regulator of energy homeostasis and FFAs oxidation [68]. It is anticipated that 50% ME of *P. niruri* might act by a similar mechanism.

Another anti-oxidant agent and bioactive compound in 50% ME of *P. niruri* is phyllanthin, which has been reported to be effective in ameliorating the development of NAFLD in mice [69]. The other possible mechanism that might contribute to the anti-NAFLD effect of 50 ME of *P. niruri* is inhibiting the activities of α-glucosidase, pancreatic lipase and cholesterol micellization. Since high rates of carbohydrate and fat consumption increase insulin resistance and oxidative stress [70], improving insulin signalling can be achieved by decreasing the intake of carbohydrates and lipids, and attenuating the postprandial hyperlipidemia. This might contribute by decreasing the hepatic cholesterol and FFAs pool required for the de novo lipogenesis process in the liver [71,72,73]. The same mechanism was also suggested for acarbose, orlistat and ezetimibe as a prospective treatment for lipid metabolic disorders such as obesity and other insulin resistance complications including NAFLD and diabetes [59,74,75]. 

The oral consumption of *P. niruri* is considered safe, and investigations into its toxicity in female Sprague–Dawley rats have not shown any abnormality in different body organs. The LD50 was higher than 5000 mg/kg body weight [76]. Moreover, it is noteworthy that *P. niruri* was clinically approved to be safely consumed by children [77]. 

**Scheme 1:**
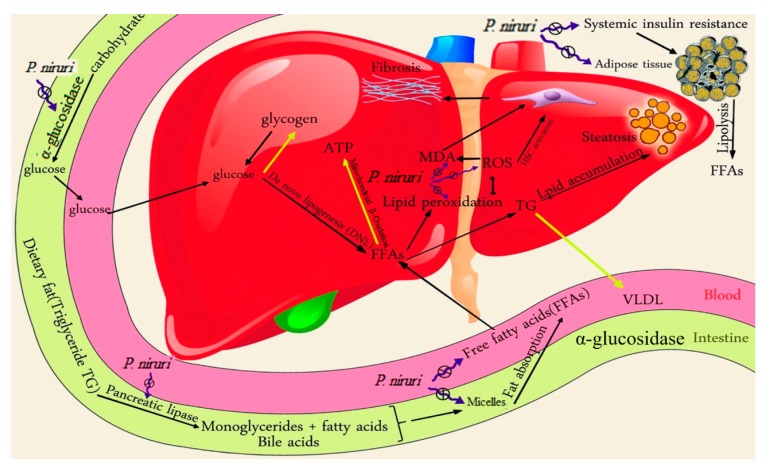
A schematic diagram simplifying the sites of action of *P. niruri* on some of the pathogenic features of NAFLD.

In summary, 50% ME of *P. niruri* reduced visceral adiposity, improved liver enzymes abnormalities, and decreased hepatic lipid peroxidation and fat accumulation. It also decreased the risk of atherosclerosis related to NAFLD, induced by a high-fat diet in SD rats. The data suggest that the antioxidant and hypolipidemic effects of the bioactive components in 50% ME of *P. niruri* (ellagic acid and phyllanthin) play essential roles in the therapeutic properties. Hence, the current study established the first-ever report on the potential therapeutic use of *P. niruri* as a natural source for treating NAFLD. The general mechanism of anti-NAFLD effect of action of *P. niruri* is shown in Scheme 1.

High dietary fat consumption combines with an increased rate of lipolysis in the insulin-resistant adipose tissues, leading to elevated free fatty acid (FFAs) concentrations in the blood. The excessive free fatty acids are delivered to the insulin-resistant liver, which has impaired mitochondrial β-oxidation. Excessive intake of carbohydrates, along with insulin resistance, result in channelling high quantities of glucose to the liver, where it is converted to either glycogen or FFAs via insulin-stimulated de novo lipogenesis (DNL), leading to excessive accumulation of triglycerides and cholesterol in the liver. *P. niruri* decreased insulin resistance, reduced serum fatty acids, and inhibited α-glucosidase, pancreatic lipase and cholesterol micellization, leading to a decrease in the amounts of glucose and FFAs in the liver. Consequently, fewer FFAs and less glucose will be available to the de novo lipogenesis process, and therefore less fat will accumulate in the liver. *P. niruri* also reduced hepatic MDA, the marker of lipid peroxidation, which stimulates hepatic stellate cells (HSC), the main cells that are responsible for triggering fibrosis in the liver. As a result, *P. niruri* reduced hepatic fibrosis. 

The yellow arrows denote the weakened pathway in NAFLD, while the black arrows refer to the stimulated pathway in NAFLD. The blue symbol indicates the inhibitory effect of *P. niruri*. 

## Figures and Tables

**Table 1:** Effect of *P. niruri* extracts and metformin on coronary risk indexes.

Parameters	NC Group	HFD Group	HFD + Met	HFD + WE	HFD + 50% ME	HFD + ME
CRI-I = TC/HDL	1.53 ± 0.03 ^b^	4.50 ± 0.57 ^d^	2.40 ± 0.10 ^b^	3.53 ± 0.26 ^d^	3.18 ± 0.16 ^a,d^	4.48 ± 0.39 ^d^
CRI-II = LDL/HDL	0.41 ± 0.05 ^b^	5.62 ± 1.10 ^d^	1.42 ± 0.19 ^b^	2.28 ± 0.24 ^b^	1.92 ± 0.17 ^b^	3.29 ± 0.44 ^a,d^
AC = (TC–HDL)/HDL	0.60 ± 0.06 ^b^	6.14 ± 1.18 ^d^	1.64 ± 0.21 ^b^	2.52 ± 0.26 ^b^	2.18 ± 0.16 ^b^	3.93 ± 0.64 ^a,d^

Values are expressed as mean ± SEM ^a^ *p* < 0.05 vs. HFD group; ^b^ *p* < 0.01 vs. HFD group; ^c^ *p* < 0.05 vs. NC group and ^d^ *p* < 0.01 vs. NC group.

**Table 2:** Grading NAFLD activity score (NAS) [47].

Steatosis	Ballooning	Lobular Inflammation	NAS
<5% (0)	None (0)	None (0)	0
5–33% (1)	Rare or few (1)	1–2 foci per 20 × field (1)	3
34–66% (2)	Many (2)	2–4 foci/20 × field (2)	6
>66% (3)	Many (2)	>4 foci/20 × field (3)	8

**Table 3:** Analysis of NAS scores for all groups.

Experimental Groups	Steatosis	Lobular Inflammation	Hepatocyte Ballooning	NAS
NC	0 ^b^	0 ^b^	0 ^b^	0 ^b^
HFD	3 ± 0 ^d^	1.83 ± 0.17 ^d^	2 ± 0	6.83 ± 0.17 ^d^
HFD + Met	1.5 ± 0.34 ^b,d^	0.67 ± 0.21 ^b^	1.83 ± 0.16 ^d^	4 ± 0.63 ^b,d^
HFD + WE	2 ± 0.26 ^a,d^	1.33 ± 0.33 ^d^	2 ± 0 ^d^	5.33 ± 0.42 ^a,d^
HFD + 50% ME	1.5 ± 0.22 ^b,d^	1 ± 0 ^a,c^	2 ± 0 ^d^	4.5 ± 0.22 ^b,d^
HFD + ME	1.67 ± 0.21 ^b,d^	1 ± 0.26 ^a,c^	2 ± 0 ^d^	4.67 ± 0.42 ^b,d^

Values are expressed as mean ± SEM ^a^ *p* < 0.05 vs. HFD group; ^b^ *p* < 0.01 vs. HFD group; ^c^ *p* < 0.05 vs. NC group and ^d^ *p* < 0.01 vs. NC group.

**Table 4:** The effect of different doses of 50% ME on NAFLD.

Parameters	NC	HFD	HFD + 50% ME		
			1000 mg/kg	500 mg/kg	250 mg/kg
Liver/body weight (%)	2.69 ± 0.21 ^b^	5.80 ± 0.11^d^	4.22 ± 0.37 ^a^	4.98 ± 0.12	4.79 ± 0.11
Visceral fat/body weight (%)	0.86 ± 0.10 ^b^	1.69 ± 0.16 ^d^	0.82 ± 0.08 ^b^	1.36 ± 0.07	1.32 ± 0.12
Glucose (mmol/L)	4.60 ± 0.27 ^a^	5.42 ± 0.26 ^c^	4.54 ± 0.11 ^b^	4.68 ± 0.10 ^a^	4.80 ± 0.10
TC (mmol/L)	1.64 ± 0.09 ^b^	7.06 ± 0.85 ^d^	4.18 ± 0.39 ^b^	4.64 ± 0.54 ^b,c^	4.76 ± 0.82 ^b,c^
LDL (mmol/L)	0.41 ± 0.02 ^b^	5.92 ± 0.81 ^d^	3.11 ± 0.34 ^b^	3.53 ± 0.49 ^b,c^	3.84 ± 3.84 ^a,d^
LDL/HDL	0.42 ± 0.03 ^b^	6.79 ± 0.57 ^d^	3.27 ± 0.34 ^a^	3.83 ± 0.45	4.54 ± 0.49
CRI-I = TC/HDL	1.66 ± 0.05 ^b^	7.40 ± 0.66 ^d^	4.38 ± 0.26 ^b^	5.26 ± 0.54 ^a,d^	5.76 ± 0.50 ^a,d^
CRI-II = LDL/HDL	0.42 ± 0.03 ^b^	6.79 ± 0.57 ^d^	3.27 ± 0.34 ^a^	3.83 ± 0.45	4.54 ± 0.49
AC = (TC − HDL)/HDL	0.65 ± 0.06 ^b^	6.99 ± 0.58 ^d^	3.37 ± 0.25 ^a^	4.27 ± 0.56	6.47 ± 0.73 ^d^
ALP (IU/L)	230.60 ± 20.18 ^a^	558.20 ± 34.75 ^c^	301.00 ± 9.54 ^a^	316.60 ± 21.76 ^a^	431.00 ± 35.05
ALT (IU/L)	67.20 ± 5.32 ^b^	107.80 ± 12.68 ^d^	65.60 ± 5.90 ^b^	70.60 ± 1.44 ^b^	59.40 ± 2.18 ^b^
AST/ALT	2.39 ± 0.17^a^	1.31 ± 0.11 ^c^	2.32 ± 0.29 ^a^	2.17 ± 0.08 ^a^	2.23 ± 0.09 ^a^
Insulin (mU/L)	13.40 ± 0.74 ^b^	44.67 ± 1.06 ^d^	14.86 ± 0.84 ^b^	15.34 ± 0.64 ^b^	19.26 ± 1.23 ^b^
HOMA-IR	2.73 ± 0.19 ^b^	10.90 ± 0.41 ^d^	2.99 ± 0.16 ^b^	3.19 ± 0.16 ^b^	4.10 ± 0.23 ^b^
Steatosis	0.00 ± 0.00 ^b^	3.00 ± 0.00 ^d^	1.20 ± 0.20 ^b^	1.40 ± 0.24 ^a^	1.80 ± 0.20
Lobular inflammation	0.00 ± 0.00 ^b^	2.00 ± 0.00 ^d^	1.00 ± 0.00 ^a^	1.20 ± 0.20	1.20 ± 0.20
Hepatocyte ballooning	0.00 ± 0.00 ^b^	2.00 ± 0.00	2.00 ± 0.00	2.00 ± 0.00	2.00 ± 0.00
NAS	0.00 ± 0.00 ^b^	7.00 ± 0.00 ^d^	4.20 ± 0.20 ^a,c^	4.60 ± 0.24 ^d^	5.20 ± 0.37 ^d^

Results are expressed as mean ± SEM, compared to HFD group. ^a^ *p* < 0.05 vs. HFD group; ^b^ *p* < 0.01 vs. HFD group; ^c^ *p* < 0.05 vs. normal group; ^d^ *p* < 0.01 vs. normal group.

**Table 5:** Anti-oxidative activities and phytochemical screening of *P. niruri* extracts.

	Total Phenolic (μg Gallic Acid Equiv./mg Extract)	Total Flavonoid (μg Quercetin Equiv./mg Extract)	DPPH IC_50_ (μg/mL)	ABTS IC_50_ (μg/mL)	FRAP (nmol Fe^+^^2^ Equiv./mg Extract)
**WE**	224.13 ± 1.14	39.41 ± 0.72	7.38 ± 0.84	31.94 ± 1.36	272.56 ± 4.39
**50% ME**	323.98 ± 0.33	64.46 ± 1.36	2.72 ± 0.25	26.51 ± 0.63	220.01 ± 1.90
**ME**	237.87 ± 0.46	49.85 ± 0.62	8.32 ± 0.29	99.66 ± 0.98	333.95 ± 11.73
**Ascorbic acid (Standard)**	---	---	1.51 ± 0.22	5.73 ± 0.09	---

Values are expressed as mean ± SEM (*n* = 3). DPPH; 2,2-diphenyl-1-picrylhydrazyl, ABTS; 2,2-azinobis (3-ethyl-benzothiazoline-6-sulfonic acid) and FRAP; ferric reducing antioxidant power.

**Table 6:** Percentage recovery, within-day and between-day precision and accuracy values for ellagic acid and phyllanthin in *P. niruri* extract.

Compounds	Concentrations (µg/mL)	Within-Day		Between-Day		Recovery	
		Accuracy (%)	Precision (CV%)	Accuracy (%)	Precision (CV%)	Mean (%)	Mean (CV%)
Ellagic acid	50.00	100.30	0.18	100.01	0.27	99.25	0.80
	6.25	97.50	0.19	97.66	2.64	-	-
	3.12	102.18	0.26	100.50	2.09	104.75	2.18
	1.56	104.36	0.51	100.64	2.83	-	-
	0.78	104.62	0.67	104.89	4.15	103.62	3.90
Phyllanthin	25.00	96.28	0.25	98.47	1.82	102.65	1.19
	12.50	104.94	1.28	102.50	3.27	-	-
	6.25	104.42	4.78	100.42	1.45	100.04	1.49
	3.12	100.83	0.28	101.10	2.63	-	-
	1.56	95.90	2.04	98.35	4.77	101.31	3.41
